# P-862. Hospital administered MOUD is associated with decreased one-year mortality among PWUD admitted with bloodstream infections

**DOI:** 10.1093/ofid/ofae631.1054

**Published:** 2025-01-29

**Authors:** Nicholas Blair, Adam Kopp, Christine J Kubin, Jesse Cotton, Michael Yin, Matt Scherer

**Affiliations:** Columbia University Medical Center, New York, New York; Columbia University Medical Center, New York, New York; NewYork-Presbyterian Hospital, New York, New York; Columbia University Medical Center, New York, New York; Columbia University Medical Center, New York, New York; Columbia University Irving Medical Center, New York, NY

## Abstract

**Background:**

Bloodstream infections (BSI) including infective endocarditis (IE) are highly morbid complications of substance use that contribute to the syndemic of invasive bacterial infections in persons who use drugs (PWUD). In this retrospective analysis we evaluated whether inpatient administration of medication for opioid use disorder (MOUD) impacted one-year all-cause mortality and antibiotic adherence in PWUD hospitalized with BSI.

**Figure:**
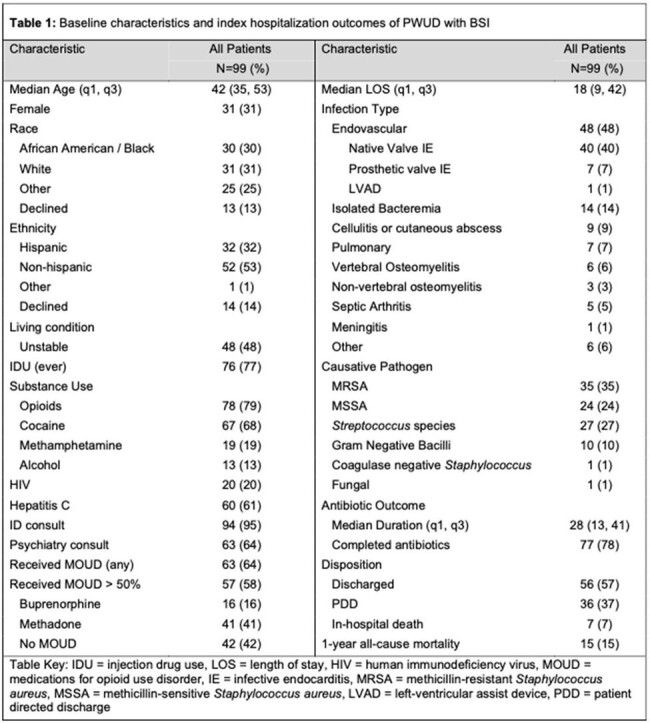

**Methods:**

We conducted a retrospective cohort study of index admissions for PWUD admitted with IE cases from 2/2020 through 7/2023 and/or BSI from 7/2022 through 7/2023 at Columbia University Irving Medical Center. Patients were included if they 1) Were aged 18-65 years old 2) Had a positive blood culture with an organism known to cause invasive disease and 3) Had documented substance use (opioid and/or stimulant) within the previous 12 months. One-year all-cause mortality was the primary outcome of interest. Administration of MOUD was counted as a treatment exposure only if received for the majority (≥ 50%) of hospitalized days. We defined the primary antibiotic outcome as a percentage of days a patient received standard of care (SOC) treatment compared to the recommended duration in days for their infection.

**Figure:**
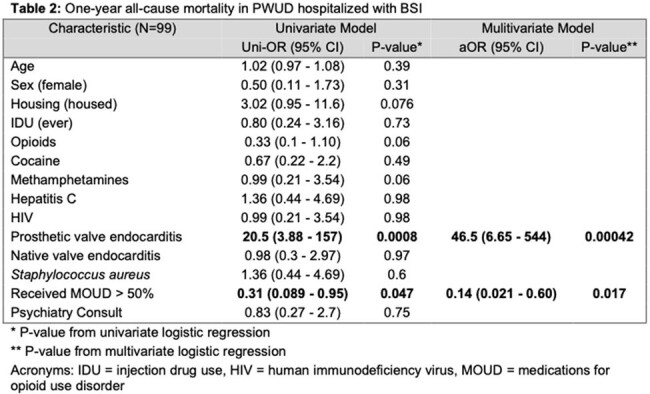

**Results:**

Of the 99 patients who met study criteria, the majority were male (69%) with a history of injection drug use (77%). Opioids (79%), cocaine (68%) and methamphetamines (19%) were the most commonly used substances (Table 1). In multivariate logistic regression, hospital administration of MOUD was associated with reduced mortality (aOR 0.14, 0.021 – 0.60, P = 0.017) after adjusting for prosthetic valve endocarditis (Table 2). In sub-analysis of the MOUD group, PWUD who received buprenorphine achieved a higher percentage of SOC antibiotic days (93%, p = 0.011) than either the methadone (61.4%) or no-MOUD (72.9%) groups (Table 3).

**Figure:**
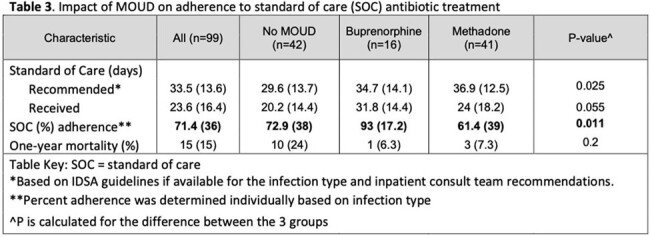

**Conclusion:**

Hospital administration of MOUD was associated with improved survival in PWUD admitted with serious bloodstream infections. Buprenorphine receipt was associated with a greater likelihood of completing SOC antibiotic course than methadone or no MOUD. To our knowledge, this is the first study to demonstrate a survival benefit to hospital administration of MOUD in PWUD during infection-related admission.

**Disclosures:**

**All Authors**: No reported disclosures

